# A Titanium (IV)–Dithiophenolate Complex and Its Chitosan Nanocomposite: Their Roles towards Rat Liver Injuries In Vivo and against Human Liver Cancer Cell Lines

**DOI:** 10.3390/ijms222011219

**Published:** 2021-10-18

**Authors:** Nadia Z. Shaban, Salah A. Yehia, Doaa Awad, Shaban Y. Shaban, Samar R. Saleh

**Affiliations:** 1 Biochemistry Department, Faculty of Science, Alexandria University, Alexandria 21515, Egypt; salah.yehia_PG@alexu.edu.eg (S.A.Y.); doaaelsayed363@alexu.edu.eg (D.A.); samar.saleh@alexu.edu.eg (S.R.S.); 2 Chemistry Department, Faculty of Science, Kafrelsheikh University, Kafrelsheikh 33516, Egypt; shaban.shaban@sci.kfs.edu.eg

**Keywords:** chitosan nanoparticles (CSNPs), titanium (IV)–dithiophenolate complex (DBT), DBT–CSNPs, liver injury, apoptosis, oxidative stress, anti-proliferative, G2/M arrest

## Abstract

Titanium (IV)–dithiophenolate complex chitosan nanocomposites (DBT–CSNPs) are featured by their antibacterial activities, cytotoxicity, and capacity to bind with DNA helixes. In this study, their therapeutic effects against rat liver damage induced by carbon tetrachloride (CCl_4_) and their anti-proliferative activity against human liver cancer (HepG2) cell lines were determined. Results of treatment were compared with cisplatin treatment. Markers of apoptosis, oxidative stress, liver functions, and liver histopathology were determined. The results showed that DBT–CSNPs and DBT treatments abolished liver damage induced by CCl_4_ and improved liver architecture and functions. DNA fragmentation, Bax, and caspase-8 were reduced, but Bcl-2 and the Bcl-2/Bax ratios were increased. However, there was a non-significant change in the oxidative stress markers. DBT–CSNPs and DBT inhibited the proliferation of HepG2 cells by arresting cells in the G2/M phase and inducing cell death. DBT–CSNPs were more efficient than DBT. Low doses of DBT and DBT–CSNPs applied to healthy rats for 14 days had no adverse effect. DBT and DBT–CSNP treatment gave preferable results than the treatment with cisplatin. In conclusion, DBT–CSNPs and DBT have anti-apoptotic activities against liver injuries and have anti-neoplastic impacts. DBT–CSNPs are more efficient. Both compounds can be used in pharmacological fields.

## 1. Introduction

The liver is the largest solid organ in the body and is required for survival. The liver has numerous functions including the synthesis of proteins, glucose, bile, and clotting factors and the breaking down of hormones, certain drugs, and xenobiotics [1,2]. Hepatic metabolism of some drugs and toxins such as carbon tetrachloride (CCl_4_) augments the generation of free radicals and reactive oxygen species (ROS), resulting in oxidative stress (OS), hepatoxicity, and deterioration of macromolecules as proteins, lipids, carbohydrates, and nucleic acids [3,4]. The hepatotoxicity induced by xenobiotics is dependent on their dosage, nature, and period of exposure [1,2]. ROS and reactive nitrogen species (RNS) are well known for playing a dual function as both harmful and beneficial species. There is increasing evidence that “double-faced” ROS in cells act as secondary messengers in intracellular signaling cascades, which stimulate and preserve the oncogenic phenotype of cancer cells, while ROS can prompt cellular senescence and apoptosis and can thus function as antitumorigenic species [5,6]. CCl_4_ is an industrial chemical found in refrigerants and solvents for waxes, varnishes, and other materials. CCl_4_ is one of the most potent hepatotoxins [5,6]. In the liver, CCl_4_ is metabolized by cytochrome P450 into the trichloromethyl radical (^•^CCl_3_), which is converted into trichloromethylperoxy radicals (CCl_3_OO^•^) [5,6].

Nanotechnology is a promising field of interdisciplinary research since it contributes to different fields, including pharmacology, parasitology, pest management, and electronics. In recent years, nanoparticles have received great attention owing to their various applications in many fields such as diagnostics, biomarkers, cell labeling [7], drug delivery, cancer therapy, and anti-flammable materials [8]. Chitosan (CS) is a natural polysaccharide and has unique characteristics frequently not detected in synthetic polymers [9]. CS nanoparticles (CSNPs) have the advantages of chitosan and the properties of nanoparticles such as surface and interface effect, small size, and quantum size effects. Therefore, the CSNPs have been reported to have key applications in ocular drug delivery, per-oral administration of drugs, brain-targeting drug delivery, parenteral drug delivery, non-viral gene delivery, vaccine delivery, mucosal drug delivery, tissue engineering, and the effective delivery of insulin [10,11].

**Figure 1:**
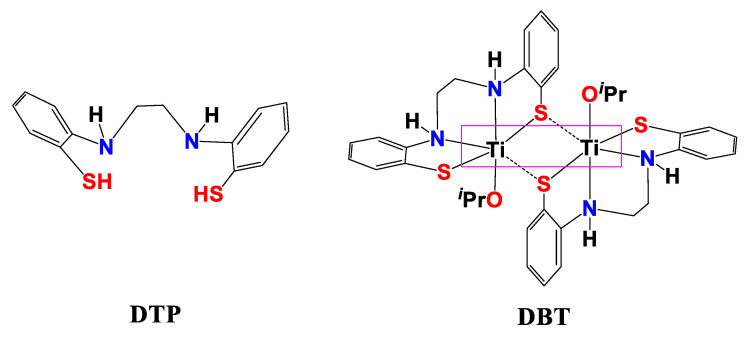
Structure of Dithiophenolato [**DTP**] and the Dithiophenolatotitanium (IV)-complex [{Ti(N_2_H_2_S_2_)(O*^i^*Pr)}x_2_] [**DBT**].

Liver diseases represent a major cause of serious and public health problems, leading to elevated levels of morbidity and mortality worldwide and in Egypt [12,13]. Chemotherapy still has a marked curative impact with substantial success in clinical practice. Although chemotherapy is an efficient way to treat many types of cancer, chemotherapy also causes serious side effects. Some chemotherapy side effects are moderate and treatable; however, others can cause dangerous complications [14]. Therefore, scientists are working to discover new treatments for liver diseases that are effective and have no/or minor side effects. Cisplatin is used to treat cancer; nonetheless, it causes serious side effects. In contrast, titanium Ti(IV) as a biomaterial is generally used in orthopedic implants such as the substitution of teeth and bone [15]. Titanium is characterized by its high effectiveness and low toxicity. In our previous studies, we prepared dithiophenolato ligand “DTP” and DTP-CS nanocomposite (DTP-CSNPs) and studied their properties. These studies revealed that these compounds can bind to DNA and have anti-bacterial and cytotoxic activities [9]. Further, DTP and DTP-CSNPs have anti-apoptotic activities against hepatotoxicity and anti-cancer towards HepG2 cell lines [16]. These results encouraged us to prepare DBT and DBT–CSNPs (Figure 1) and study some of their biological properties. Our studies showed that these compounds have an ability to bind to DNA and have anti-bacterial and cytotoxic activities [17]. We are interested in this type of thiophenolato compound because it contains the thiolate S donors that have π-acceptor ability and can tune the electron density at the chitosan moiety through σ donor and π back-bonding characters [18]. It was found that the titanium center enhances the DBT affinity for DNA and changes the binding mechanism. When DBT and its Ti (IV) complex were included in CSNPs, the Ti (IV) center was found to reduce the affinity but not change the mechanism. These results showed that the metal center may not always be important for binding. In this regard, the current study attempted to investigate the therapeutic effect and demonstrated a very important issue, that the metal center may have an important therapeutic effect. Therefore, after the determination of LD_50_ (the lethal dose: the amount of a compound that causes the death of 50% of a group of test animals) values of DBT and DBT–CSNPs, the therapeutic effect of DBT and DBT–CSNPs against rat liver injury induced by CCl_4_ was investigated in detail. The study focused on the determination of the markers of OS, apoptosis, liver functions, lipid profile, and kidney functions besides liver histopathology. Additionally, the effect of these compounds on cell cycle analysis in normal liver (THLE2) and HepG2 cell lines was investigated to determine if these compounds have antitumor activity.

## 2. Results

### 2.1. Thermal Stability of DBT and DBT–CSNPs

The data revealed that DBT–CSNPs have a spherical morphology where the particle size was about ~85 ± 2 nm. The CS nanocomposite has a spherical shape and an average particle size of 75 ± 3 nm.

### 2.2. LD_50_ of DBT and DBT–CSNPs

The current results showed that the LD_50_ values of DBT and DBT–CSNPs were about 1350 mg/kg and 1800 mg/kg, respectively (Table 1).

### 2.3. Effect of Treatments with DBT, DBT–CSNPs, and Cisplatin on CCl_4_-Induced Hepatotoxicity

#### 2.3.1. Effect of Different Studied Compounds on OS Markers

**Figure 2:**
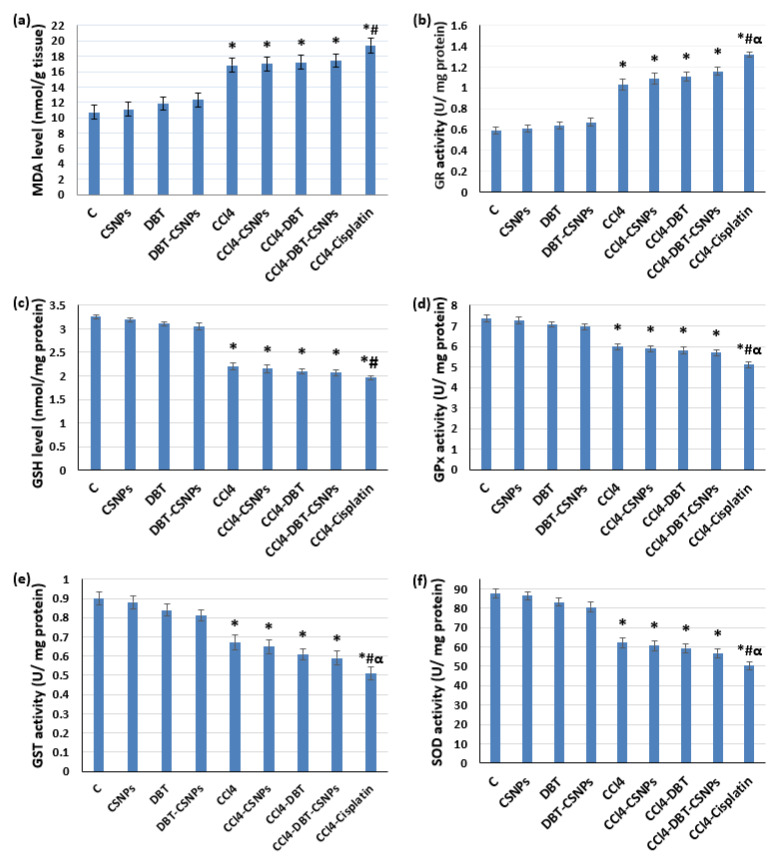
**Effect of different studied compounds on OS parameters.** (**a**) malondialdehyde (MDA), (**b**) glutathione reductase (GR), (**c**) reduced glutathione (GSH), (**d**) glutathione peroxidase (GPx), (**e**) glutathione-S-transferase (GST), and (**f**) superoxide dismutase (SOD) activities. The values represent the mean ± SD of eight rats. One-way ANOVA was used (* *p* < 0.05 versus saline control group, # *p* < 0.05 versus CCl_4_ group & α *p* < 0.05 versus DBT–CSNP-treated group).

The present results revealed that CCl_4_ injection caused significant (*p* < 0. 05) elevations in the malondialdehyde (MDA) level and glutathione reductase (GR) activity associated with a significant (*p* < 0.05) decrease in the glutathione (GSH) level and the activities of total glutathione peroxidase (GPx), glutathione-S-transferase (GST), and superoxide dismutase (SOD) as compared to the control group (Figure 2).

In contrast, treatment with DBT and DBT–CSNPs (for 14 days) after CCl_4_ injection caused non-significant changes (increase or decrease) in the oxidant and antioxidant markers (MDA and GSH levels, beside GPx, GST, and SOD activities) as compared to the CCl_4_ group. Further, the results showed that CSNP treatment after CCl_4_ injection caused non-significant changes in the oxidant and antioxidant markers when compared to the CCl_4_ group. However, treatment with cisplatin (for 4 days) after CCl_4_ injection significantly increased MDA levels; nevertheless, it significantly decreased the antioxidant markers. Administration of CSNPs, DBT, and DBT–CSNPs to healthy rats (for 14 days) caused non-significant changes in the oxidant and antioxidant markers (Figure 2).

#### 2.3.2. Effect of Different Studied Compounds on Apoptosis

**Figure 3:**
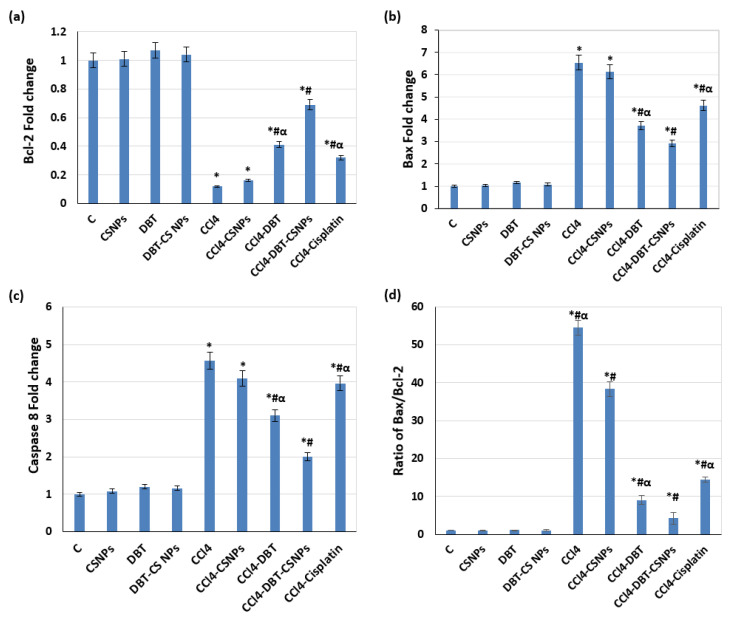
**Effect of different compounds on apoptotic markers.** (**a**) The mRNA levels of Bcl-2. (**b**) The mRNA levels of Bax. (**c**) mRNA levels of caspase-8. (**d**) Relative ratio of Bax/Bcl-2 mRNA. Gene expression was normalized to β-actin. The data are expressed as the mean ± SD of three rats. One-way analysis of variance (ANOVA) was used (* *p* < 0.05 versus saline control group, # *p* < 0.05 versus CCl_4_ group & α *p* < 0.05 versus DBT–CSNP-treated group).

**Figure 4:**
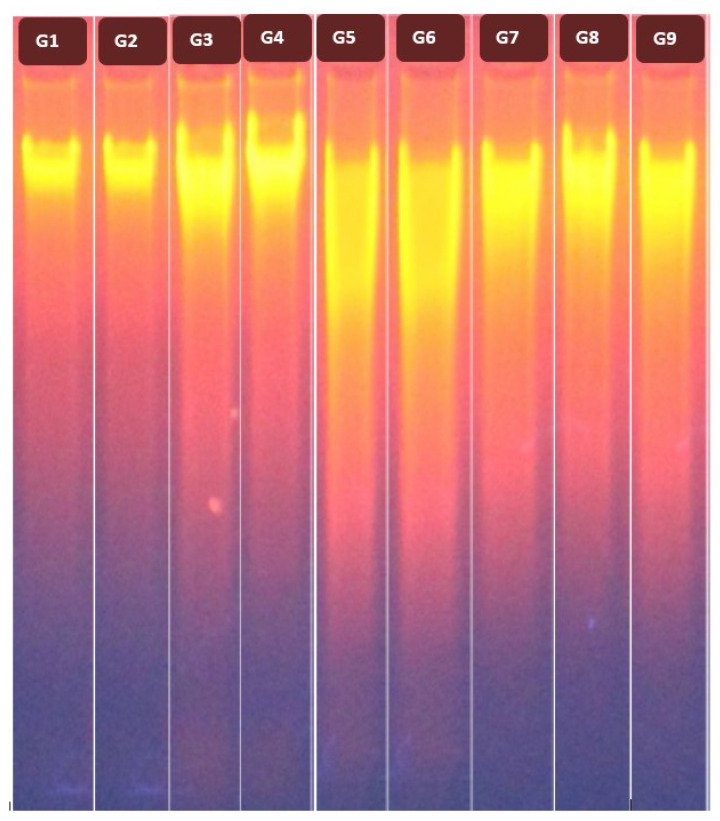
**Effect of different compounds on DNAF.** Ethidium bromide-stained agarose gel showing fragmentation patterns of the extracted DNA in the studied groups. G1: control, G2: CSNPs, G3: DBT, G4: DBT–CSNPs, G5: CCl_4_, G6: CCl_4_-CSNPs, G7: CCl_4_-DBT, G8: CCl_4_-DBT–CSNPs, and G9: CCl_4_-cisplatin.

The current results revealed that CCl_4_ administration caused a significant (*p* < 0.05) down-regulation of Bcl-2 expression with significant (*p* < 0.05) up-regulations of Bax and caspase-8 expressions as compared to the control group (Figure 3a–c). Furthermore, a significant elevation (*p* < 0.05) in the ratio of Bax/Bcl-2 was detected in rats after CCl_4_ injection compared to the control group (Figure 3d). Further, CCl_4_ administration increased DNA fragmentation (DNAF) significantly (*p* < 0.05) as compared to the control group (Figure 4, lane 5). In contrast, treatment with DBT and DBT–CSNPs and cisplatin after CCl_4_ injection caused a significant (*p* < 0.05) elevation in the Bcl-2 expression level with a significant (*p* < 0.05) decline in the expression levels of Bax and caspase-8, Bax/Bcl-2 ratio, and DNAF as compared to the CCl_4_ group (Figure 3 and Figure 4). In addition, treatment with CSNPs after CCl_4_ injection caused non-significant (*p* < 0.05) changes (increase or decrease) in the levels of apoptotic markers (BCl-2, Bax, and caspase-8 expressions as well as the DNAF) with a significant decrease in Bax/Bcl-2 ratio compared to the CCl_4_ group (Figure 3 and Figure 4). On the contrary, the administration of CSNPs, DBT, and DBT–CSNPs in healthy rats caused non-significant changes in the levels of apoptotic markers compared to the control group (Figure 3 and Figure 4).

### 2.4. Effect of Different Studied Compounds on Liver and Kidney Functions and the Lipid Profile

The results (Table 2) showed that CCl_4_ administration caused significant (*p* < 0.05) elevations in the activities of serum alanine aminotransferase (ALT), aspartate aminotransferase (AST), and alkaline phosphatase (ALP), but it substantially reduced the levels of serum albumin, serum total protein (STP), and liver total protein (LTP) compared to the control group. Further, the levels of serum cholesterol, triglyceride (TG), and low-density lipoprotein (LDL) were also significantly increased (*p* < 0.05); nonetheless, the serum high-density lipoprotein (HDL) level was significantly decreased (*p* < 0.05) (Table 2). Additionally, CCl_4_ induced nephrotoxicity where serum urea and creatinine levels were increased significantly (*p* < 0.05, Table 2) when compared with the control group. In contrast, treatment with DBT and DBT–CSNPs after CCl_4_ administration improved liver functions, the lipid profile, and kidney functions to different degrees (Table 2). Treatment with cisplatin after CCl_4_ improved liver function, the lipid profile, and kidney functions as well as the lipid profile, but to a lesser extent than in those treated with DBT and DBT–CSNPs (Table 2). Treatment with CSNPs after CCl_4_ administration non-significantly improved liver, kidney functions, and lipid profiles as compared to the CCl_4_ group. Otherwise, administration of healthy rats with CSNPs, DBT, and DBT–CSNPs, separately, for 14 days induced non-significant changes in the liver and kidney function and lipid profiles as compared to the control group (Table 2).

### 2.5. Histopathological Analysis

**Figure 5:**
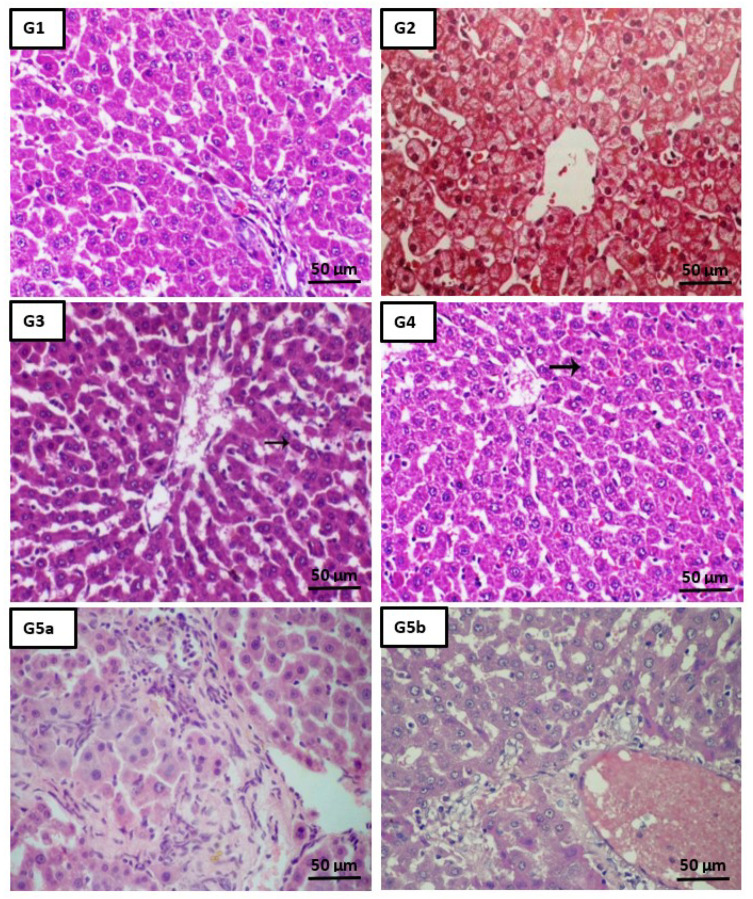

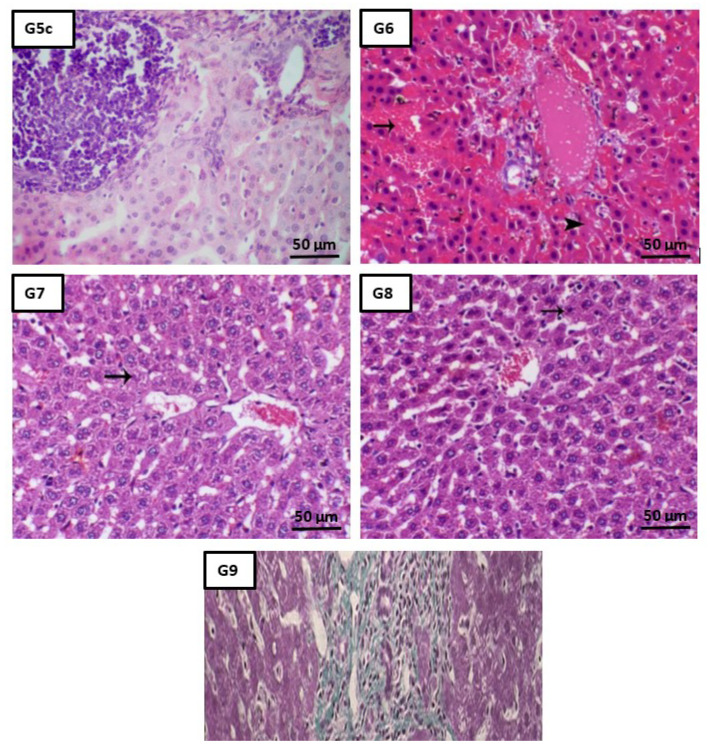
**Effect of different studied compounds on the liver histology.** Liver tissues from the control group (G1: H&E, ×200, Scale bar = 50 µm) showed a normal lobular architecture, individual hepatocytes disclosed no pathology, and the portal triad was undetected. Liver tissues of groups CSNPs, DBT, and DBT–CSNPs (G2–G4, respectively: H&E, ×200, Scale bar = 50 µm) exhibited normal hepatocytes around the central vein. A tissue sample from the intoxicated CCl_4_ group (G5: H&E, ×200, Scale bar = 50 µm) revealed cellular infiltration, congestion of central vein, mild portal inflammation, hemorrhage as well as centrilobular hepatic necrosis, and focus of lytic necrosis & dispersed apoptotic bodies both intra & extracellular in location. The specimen of CSNP-treated rats (G6: H&E, ×200, Scale bar = 50 µm) demonstrated congestion of the central vein and hemorrhage (arrow) and centrilobular hepatic necrosis (arrowhead). The specimen of DBT-treated rats (G7: H&E, ×200, Scale bar = 50 µm) confirmed a mild degree of hepatic degeneration represented by cell swelling (arrow). On the contrary, the liver tissue of DBT–CSNP-treated rats (G8: H&E, ×200, Scale bar = 50 µm) revealed a marked decrease in hepatic degeneration unless single cell degeneration (arrow). Moreover, a periportal inflammatory reaction with a degenerated hepatic cord and disrupted cell plates was observed in the tissue of the cisplatin-treated group (G9: H&E, ×200, Scale bar = 50 µm). These histopathological results revealed the hepatoprotective effects of DBT and DBT–CSNPs, confirming the biochemical analysis.

Figure 5 (G1–G9) shows the histopathological examination of liver tissues of different studied groups. Treatment with both DBT and DBT–CSNPs after CCl_4_ injection improved liver histopathology caused by CCl_4_, elucidating the therapeutic roles of DBT and DBT–CSNPs that emphasize the biochemical analysis. However, cisplatin treatment after CCl_4_ improved liver histopathology caused by CCl_4_ but to a degree less than the treatment with DBT or DBT–CSNPs. Administration of CSNPs, DBT, and DBT–CSNPs, separately, to the healthy rats had no effect on normal liver architecture.

### 2.6. DBT and DBT–CSNP-Induced Cell Cycle Arrest

**Figure 6:**
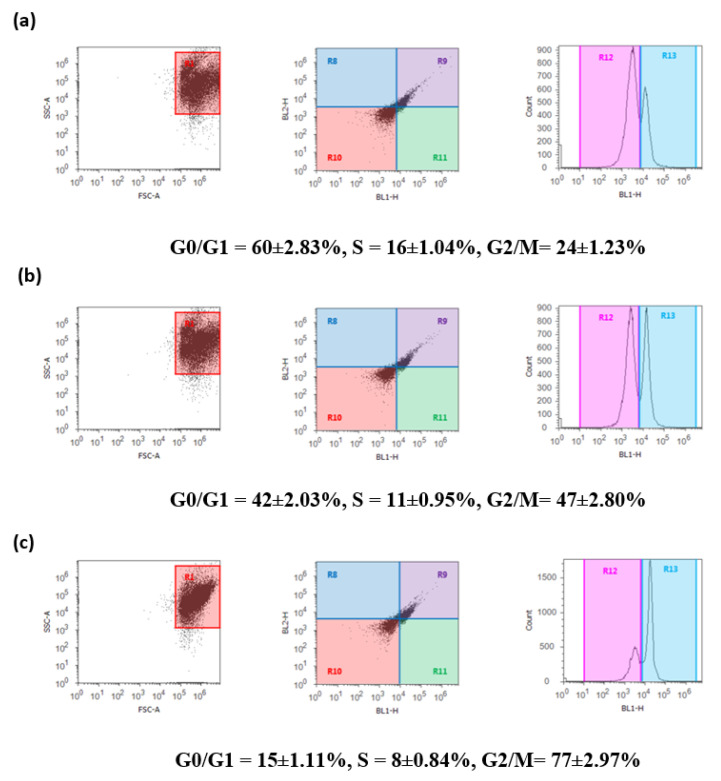

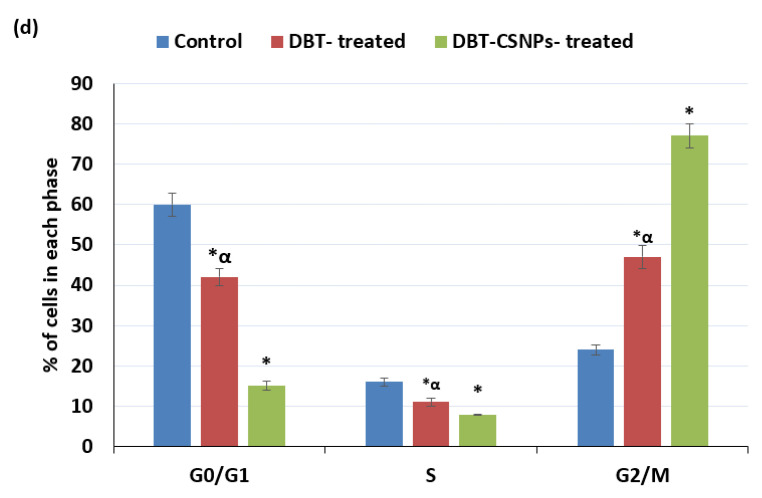
**Flow cytometric analysis of control and treated HepG2 cells.** (**a**) Control, (**b**) DBT-treated HepG2 cells, and (**c**) DBT–CSNP-treated HepG2 cells. The values represent the mean ± SD (*n* = 3). (**d**) Represents % of cells in each phase. One-way ANOVA followed by Tukey’s test was used (* *p*< 0.05 versus saline control & α *p* < 0.05 versus DBT–CSNPs).

The current results showed that treatment of HepG2 cells with DBT and DBT–CSNPs caused a significant decrease in the population of HepG2 cells in the G0/G1 and S phases when compared with normal cells (Figure 6). Additionally, high populations of HepG2 cells were halted at G2/M checkpoint compared to the untreated cells. Further, the data showed that the cells treated with DBT–CSNPs showed the lowest levels in the G0/G1 and S phases with the highest in the G2/M phase as compared to those treated with DBT (Figure 6).

## 3. Discussion

DBT–CSNPs have a spherical morphology with an average particle size of ~85 ± 2 nm, while the CS nanocomposite has a spherical shape and an average particle size of 75 ± 3 nm. The presence of DBT in the DBT–CSNPs was found to increase the thermal stability of the composite material in comparison to DBT [17]. In our previous studies, the TEM images revealed the compatibility of DBT–CS surface adsorption through the time of reaction. This interaction may be related to the hydroxyl groups that are present on the surface of DBT, as these hydroxyl groups may form hydrogen interactions with the amino groups at certain sites along the CS nanoparticles. Furthermore, the SEM spectrum images confirmed the TEM morphology and showed that DBT–CS particles are linked as bundles with a leaf structure. Further, energy-dispersive X-ray (EDAX) analyses revealed the presence of the NK, OK, SK, and TiK atoms in the elemental composition of the prepared nanocomposite [17]. The DBT–CS nanocomposite has major CS nanoparticle components and a minor amount of DBT.

Additionally, the LD**_50_** values of DBT and DBT–CSNPs were about 1350 and 1800 mg/kg, respectively. Previous studies revealed that the LD_50_ values of oral titanium tetrachloride and oral titanium oxide nanoparticles (TiO_2_ NPs) were 1780 mg/kg and greater than 12 g/kg, respectively [19]. The LD_50_ values of DTP and CSNP-DTP were about 2187.5 and 1462.5 mg/kg, respectively [16]. In this study, the rat hepatotoxicity induced by CCl_4_ administration was treated with safe doses of DBT and DBT–CSNPs (4.5 and 3 mg/kg BW, respectively). The doses of DBT and DBT–CSNPs were chosen to be close to the dose of cisplatin, which is given to patients (4 mg/kg BW). Generally, cisplatin is given for four days only, but it causes side effects, while DBT and DBT–CSNPs are given for a long period (14 days) since titanium is distinguished by its nontoxicity.

The present study demonstrated that repeated administration with CCl_4_ induced hepatotoxicity and liver damage via the elevation of OS and apoptosis. This led to the disturbance in liver functions as liver enzymes were elevated in the blood circulation and the lipid profile changed. These results are in harmony with the histological results. In contrast, treatment of rats with DBT and DBT–CSNPs after CCl_4_ mitigated the hepatotoxicity and liver damage to different degrees through the reduction of apoptosis caused by CCl_4_, which improved liver functions, the lipid profile, and liver histopathology. Interestingly, the DBT–CSNP treatment showed better effects than treatment using DBT and cisplatin, which is used as a standard drug. All these conclusions are discussed in detail as follows. The data of the current study demonstrated that CCl_4_ administration induced hepatotoxicity, resulting in severe liver damage as revealed from the histopathological results, which were confirmed by the biochemical results comprising the markers of liver functions, the lipid profile, and apoptosis in liver tissues. The mechanism of CCl_4_-induced hepatotoxicity may be related to the harmful effect of CCl_4_ and its extremely reactive metabolites (CCl_3_* and CCl_3_O_2_*). These free radicals induced OS as demonstrated from the change in the MDA level and GR activity with lower levels of the antioxidants (GSH, GPx, GST, and SOD). MDA is the main product of peroxidation of polyunsaturated fatty acids and its elevated content is a significant indicator of lipid peroxidation [20].

The elevation in the peroxidation of the mitochondrial membrane increased membrane permeability and altered calcium homeostasis, resulting in the loss of cell integrity that contributed to cell death [5,6]. GR plays a key role in cellular defense against OS by preventing the accumulation of oxidized glutathione (GSSG) and thus maintaining the redox state. Therefore, the increase in GR activity after CCl_4_ administration possibly reflects an adaptation to oxidative condition and this agrees with a previous study [1]. In contrast, free radical scavengers (GSH, GPx, GR, GST, and SOD) protect the biological systems from the deleterious effects of free radicals [21]. GSH plays an important role against lipid peroxidation induced by CCl_4_ through covalent binding to ^•^CCl_3_ and CCl_3_OO^•·^radicals [22]. Additionally, GSH acts as a cofactor for GPx and as a nucleophilic scavenger of numerous compounds [22]. In this study, depletion of the GSH level after CCl_4_ administration could contribute to the stimulation of lipid peroxidation [2,22].

Additionally, SOD is the body’s first line of protection against superoxide radicals [19,20], where it catalyzes the dismutation of the superoxide radical into ordinary molecular oxygen and H_2_O_2_ [20]. GPx, a selenium-containing enzyme, is the second line of protection against hydroperoxides by catalyzing the reduction of H_2_O_2_ and lipid peroxides, in the presence of GSH, to water and lipid alcohols, respectively, while GSH is transformed into GSSG [2,22]. Furthermore, GSTs are considered as major phase II detoxification enzymes and are mainly present in the cytosol since they catalyze the conjugation of GSH with a wide range of electrophilic substances. Subsequently, the reduction in the activities of antioxidant enzymes may be related to their inhibition by CCl_4_ and its reactive metabolites through direct interaction with the enzyme molecules. Further, GSH depletion led to the inhibition of GPx and GST. Additionally, SOD inhibition resulted from the oxidation of the cysteine residues in its molecules [3,23].

Apoptosis is a form of cell death where the programmed concatenation of the process results in the removal of superfluous cells without releasing deleterious substances into the surrounding area [24]. Apoptosis is tightly regulated by particular genes, including several pro-and anti-apoptotic proteins [24,25]. Proteins of the Bcl-2 family exert different effects; for example, Bcl-2 and Mcl-1 are anti-apoptotic proteins, while the white B-cell lymphoma X protein (Bax, Bad and Bak) exhibits pro-apoptotic effects. Dysfunction of apoptosis renders the cancer cell to become resistant to treatment as well as promoting tumorigenesis [25,26]. Bax activation elicits cytochrome-c release, procaspase-3 activation, and Poly (ADP-ribose) polymerase cleavage through the stimulation of Apaf-1 (apoptosis protease activating factor-1), which leads to the induction of the apoptosis response and cell death [27]. Caspase-8 is a cysteine protease that initiates apoptotic signaling via the extrinsic pathway. Activation of caspase-8 induces apoptosis through the activation of caspase-3 directly or activation of Bax, which in turn activates caspase-3, resulting in the cleavage of essential substrates for cell viability, inducing cell death [27,28]. Furthermore, cleavage of chromosomal DNA into oligonucleosomal size fragments is an essential part of apoptosis [24,25,29].

The current results showed that CCl_4_ administration induced the regulation of pro-apoptotic proteins Bax and caspase-8, with down-regulation of anti-apoptotic protein Bcl-2. Changes in Bcl-2 and Bax levels stimulated a change in the ratio of Bax/Bcl 2 that became much higher than that of the control group. Elevation in the Bax/Bcl-2 ratio caused permeabilization of the outer membrane of the mitochondria, releasing cytochrome C into the cytoplasm [4,30]. Furthermore, the elevation in caspase-8 after CCl_4_ administration stimulated apoptosis via the activation of caspase-3 directly or indirectly [27,29].

Moreover, the elevation in ROS after CCl_4_ administration induces the elevation of p53 signaling, which in turn stimulates Bax expression; nonetheless, it blocks Bcl-2 expressions (i.e., the ratio of Bax/Bcl-2 was elevated), leading to apoptosis [31,32]. In addition, the current results revealed that CCl_4_ induced apoptosis via the induction of DNAF where its level was increased significantly compared to the control group. This may be related to the reaction of •CCl_3_ and CCl_3_OO• and other ROS with DNA, forming adducts [1,23] that activated pro-apoptotic factors, promoting apoptosis and cell death [1,2,33]. These results are consistent with previous studies, which demonstrated that CCl_4_ induced rat liver fibrosis through the activation of apoptosis [4,30,34].

In general, the present results showed that CCl_4_ administration induced OS and apoptosis, and this led to acute liver injuries wherever these results were in unison with the histopathological results. Additionally, liver injuries after CCl_4_ administration were assured by the elevation of the activities of liver enzymes (AST, ALT, and ALP) and the lipid profile (TG, TC, and LDL cholesterol) in serum with a decrease in albumin, LTP, STP, and HDL cholesterol.

In contrast, treatment of rats with DBT and DBT–CSNPs (for 14 days) after CCl_4_ administration caused an amelioration of the hepatic histopathology as revealed by the liver architecture. These results are evidenced by the amelioration of liver functions and lipid profile. The levels of ALT, AST, and ALP became lower than those in CCl_4_ group, while LTP and STP were improved. Further, the levels of TG, total cholesterol, and LDL cholesterol became lower than those of the CCl_4_ group, while HDL cholesterol became greater. Additionally, the renal functions were improved as shown by the levels of urea and creatinine, which became lower than those of the CCl_4_ group. All these positive results can be attributed to the therapeutic effects of DBT and DBT–CSNPs, which diminished liver injuries induced by CCl_4_ via the reduction of apoptosis induced by CCl_4_ as demonstrated by the current results. First, treatment with DBT and DBT–CSNPs caused non-significant changes in the OS in the liver as compared to the CCl_4_ group, where MDA as an oxidant and GR activity were insignificantly increased; nevertheless, the antioxidants (GSH, GPx, GST, and SOD) were insignificantly decreased. Non-significant changes in OS induced by these treatments did not adversely affect the liver as shown by the results of liver functions, the lipid profile, and liver histopathology as mentioned above, but improved them. The non-significant elevation in the MDA level may be related to the metabolites of the DBT and DBT–CSNPs (TiO_2_ and TiO_2_NPs, respectively). TiO_2_ and TiO_2_NPs promote the generation of ROS such as –O_2_• and H_2_O_2_ [35]. Interestingly, the previous studies showed that the thiol groups in the DBT and DBT–CSNPs play an important role in the reduction of H_2_O_2_. The thiol groups are oxidized in the presence of H_2_O_2_ formation (O-S-O) leading to the non-significant elevation in the MDA level compared with CCl_4_ [36]. On the contrary, SOD and GPx are zinc- and selenium- dependent enzymes, respectively. Therefore, the reduction in their activities in the present study may be due to the replacement of zinc and selenium ions with Ti ions, which resulted in the inhibition of these enzymes. Further, Ti ions may bind with the sulfhydryl groups of GSH, resulting in diminished GSH levels and thus the inhibition of GPx and GST activities [37]. In general, the results revealed that DBT–CSNPs had a greater effect than DBT and this may be due to the physicochemical properties of the nanoparticles that are distinguished by a nano-size, resulting in increased surface area/unit mass and the surface property effects facilitating their passage through cell membranes hydrolyzed into TiO_2_NPs and interruption of the biological systems [36,37,38,39]. The current results are consistent with the previous studies, which showed that the nanoparticles motivate OS, and this process is dose-dependent [40,41].

The present results revealed that treatment of rats with cisplatin for 4 days after CCl_4_ injection significantly increased the OS as demonstrated by the elevation of lipid peroxidation and reduction of antioxidant parameters leading to liver damage, resulting in the elevation of serum liver enzymes and changing the lipid profile. These results coincide with previous studies [42,43,44].

The administration of CSNPs, DBT–CSNPs and DBT to healthy rats for 14 days caused non-significant changes in the levels of OS markers (MDA, GR, GSH, GPx, GST, and SOD) when compared to the control group, leading to non-significant effects in the markers of the lipid profile and liver and kidney functions. Although DBT–CSNPs had a greater effect than DBT, they did not change the liver histology.

On the contrary, treatment of rats with DBT and DBT–CSNPs, as well as cisplatin after CCl_4_ caused a significant (*p* < 0.05) elevation in the Bcl-2 expression level with a significant (*p* < 0.05) decline in the levels of DNAF, Bax, and caspase-8 expressions besides the Bax/Bcl-2 ratio as compared to the CCl_4_ group. The reduction in the Bax/Bcl-2 ratio led to the stabilization of the mitochondrial membrane and prevented the release of cytochrome C, resulting in the lowering of apoptosis. The decreased Bax/Bcl-2 ratio led to the stabilization of the mitochondrial membrane and prevented the release of cytochrome C, resulting in diminished apoptosis.

In addition, the administration of rats with CSNPs only after CCl_4_ injection induced non-significant changes (increase or decrease) in all markers of apoptosis as compared to the CCl_4_ group, indicating that DBT–CSNP treatment after CCl_4_ was mainly related to the effect of DBT not CSNPs. Interestingly, treatment with DBT–CSNPs showed a greater effect than the DBT treatment, and this may be due to the variation in the physicochemical properties of these compounds and their metabolites as previously mentioned.

The results of the present study agree with the previous studies, which reported that TiO_2_NPs have anti-apoptotic action greater than TiO_2_, and their effects are dose-, period-, and cell type-dependent [30].

Moreover, treatment with DBT and DBT–CSNPs for 14 days after CCl_4_ gave better results than the treatment with cisplatin (dose) for four days (the period of treatment for cancer patients with this drug). As indicated by the current results, cisplatin treatment significantly increased OS, lipid peroxidation, and liver damage, resulting in the elevation of serum liver enzymes and changing the lipid profile and kidney functions. All these adverse effects that resulted from cisplatin treatment are in accordance with previous studies [45,46]. In contrast, 14-day DBT and DBT–CSNP treatment increased the OS non-significantly, and thus there were non-significant changes in the liver functions, lipid profile, and kidney functions.

Moreover, treatment with DBT and DBT–CSNPs for 14 days after CCl_4_ gave better results than the treatment with cisplatin (dose) for four days. As indicated by our results, cisplatin treatment significantly increased OS and lipid peroxidation as compared with CCl_4_, illustrating that the total lipid peroxidation was caused by CCl_4_ and cisplatin. Therefore, treatment with DBT and DBT–CSNPs improved the liver functions and lipid profile and kidney functions.

However, cisplatin treatment improved liver functions and the lipid profile, but to a lesser degree than DBT and DBT–CSNPs. Further, cisplatin treatment caused non-significant improvement in kidney functions compared to the CCl_4_ group. These results agree with the previous studies, which showed that the clinical use of cisplatin is strictly limited, particularly by dose-dependent side effects [1,30]. Although cisplatin is one of the majority effective chemotherapeutic drugs for the treatment of different cancers [47], it causes nephro-, neuro-, cardio-, and hepatotoxicity due to its accumulation in the liver, kidneys, and other organs, generating free radicals resulting in OS, and thus inducing damage to the liver and kidneys, as well as different organs [43,48]. Furthermore, the results showed that the administration of DBT, DBT–CSNPs, or CSNPs to healthy rats caused non-significant changes in apoptosis, where all studied markers were non-significantly changed as compared to the control group. 

DBT and DBT–CSNPs are featured by their antitumor activities against HepG2 cells. Cell growth and proliferation are involved in cell cycle organization, and an imbalance between them induces apoptosis that is implicated in the growth and progression of most tumors [47]. Our previous study showed that DBT and DBT–CSNPs have cytotoxic effects, and they can link to DNA. Therefore, the present study attempted to evaluate their anti-proliferative effect toward the HepG2 cell line through cell cycle analysis to determine whether these compounds have antitumor activities. Consequently, the present results revealed that treatment with DBT and DBT–CSNPs caused a significant decrease in the population of HepG2 cells in the G0/G1 and S phases when compared to the normal cells. This elucidates that these compounds overlapped with DNA synthesis and disrupted the advancement of the cell cycle, resulting in apoptosis. Moreover, these compounds arrested high populations of HepG2 cells at the G2/M checkpoint when compared with untreated cells. This indicates that the cell cycle arrest resulted in disruption of the tubulin-microtubule equilibrium and allowed the time for DNA repair or allowed cells to survive through persistent DNA damage. Generally, DBT and DBT–CSNPs have antitumor activities involved in cell division and stopping the uncontrolled proliferation of cancer cells besides initiating apoptosis, and this is considered to be a significant strategy. The antiproliferative and apoptotic activities of DBT and DBT–CSNPs perhaps may be due to the cytotoxicity of these complexes and their metabolites TiO_2_ and TiO_2_NPs, respectively. Previous studies showed that TiO_2_NPs induce OS in HepG2 cells, DNAF, and p53 activation, leading to apoptosis [38,40]. Further, it has been reported that a drug delivery system promotes clinical conclusions by enhancing permeability and its rate, qualifying targeted delivery, and simple distribution of the drug, leading to amelioration of the efficacy of several drugs [49].

In general, our results revealed that DBT–CSNPs and DBT treatments decreased rat liver apoptosis induced by CCl_4_, resulting in the improvement of liver architecture and functions besides reducing nephrotoxicity. In addition, DBT–CSNPs and DBT had anticancer activities against HepG2 cell lines through the prevention of their proliferation by increasing apoptosis via arresting the cell cycle in the G2/M phase. These results indicate that the DBT–CSNPs and DBT are selective, and this means that they have an ability to distinguish between liver injuries and cancer cell lines. The current results agree with the previous studies, which reported that the effect of drugs and xenobiotics in cell lines and in vivo are different from each other [50,51].

## 4. Materials and Methods

### 4.1. Chemicals

Bound HepG2 was obtained from the American Type Culture Collection (ATCC, Manassas, VA, USA). GIBCO^®^ Minimum Essential Medium (MEM) was purchased from GIBCO (Grand Island, New York, NY, USA). Dulbecco’s Phosphate-Buffered Saline medium and L-glutamine were obtained from Invitrogen (Carlsbad, CA, USA). Trypsin-EDTA, penicillin, and streptomycin were purchased from Thermo Fisher Scientific (Waltham, MA, USA). Cisplatin, CCl_4_, Dimethyl sulfoxide (DMSO), and other analytical-grade chemicals were purchased from Sigma Aldrich (St. Louis, MO, USA). The kits for the methyl thiazolyl tetrazolium (MTT) assay and RNA extraction were obtained from Eugene, Oregon, USA, and Thermo Scientific, Fermentase, respectively. All other analytical grade chemicals were obtained from Sigma (St. Louis, MO, USA) and Merck (Darmstadt, Germany). All dilutions were made with high-purity deionized water, obtained from a Milli-Q^®^ system (Merck Chemicals GmbH, Darmstadt, Germany).

### 4.2. Animals

Adult male Sprague–Dawley rats (number: 120 and weighing 110–140 g) were obtained from the experimental animals Breeding Centre of the Holding Company for Biological Products and Vaccines (Helwan, Cairo, Egypt). They were housed in stainless cages under standard laboratory conditions of 50 ± 5% air humidity, a 12-h light/dark cycle, and at a room temperature of 23 ± 2 °C. Rats received a standard laboratory diet and tap drinking water and were left for two weeks as an adaptation period. All animal methodology followed the Institutional Animal Care and Use Committee (IACUC) and was approved through the Committee of the Animal Care and Use at Alexandria University (Ethical approval reference number: AU 04 20 06 20 2 02, approved on 20/06/2020).

### 4.3. Preparation of DBT–CSNPs

**DBT** [Ti(N_2_Me_2_S_2_)(O^i^Pr)_2_] was synthesized from the thiolate-amine ligand N_2_Me_2_S_2_^2−^, containing the N,N′-dimethylethylenediamine backbone and titanium tetra (iso-propoxide) in methanol as a solvent. The crystal structure exhibited twisted octahedral geometry with respect to titanium with the two isopropoxo groups being cis to each other and to the two tetradentate ligand thiolate groups. The ligand’s two tertiary amine atoms are cis to each other and essentially trans to terminal oxo groups [18]. CSNPs were prepared using ionic gelation techniques using chitosan and sodium tripolyphosphate (TPP). DBT–CSNPs were prepared from DBT, sodium tripolyphosphate, and chitosan [17]. In brief, sodium tripolyphosphate solution was added to the chitosan solution, left at 25 °C for 12 h, after which DBT was added, left for 40 min, and the solvent was removed at 40 °C. Characterization of DBT–CSNPs was examined by a High-Resolution Transmission Electron Microscope (HR-TEM), Scanning Electron Microscope (SEM) with an EDX detector, X-Ray Diffraction (XRD), Fourier transform infrared (FT-IR), and Thermographymetric Analysis (TGA) [17].

### 4.4. Determination of the LD_50_ Values of DBT and DBT–CSNPs

Initially, the LD_50_ values of DBT or DBT–CSNPs were estimated mathematically using the values of IC50 (μg/mL) according to the regression formula obtained from the Interagency Coordinating Committee on the Validation of Alternative Methods: (ICCVAM) log LD_50_ (mg/kg) = 0.372 logs IC_50_ (μg/mL) + 2.024 [52]. The LD_50_ values obtained theoretically facilitate the determination of LD_50_ in vivo. For determination of the LD_50_ of DBT and DBT–CSNPs, 48 rats were divided into 12 groups, and six doses of DBT and DBT–CSNPs, separately, were dissolved in 2% DMSO (200, 400, 800, 1200, 2000, and 3000 mg/kg) and were used for intraperitoneal (IP) injection at once. Animals were examined for any abnormal clinical signs and behavioral changes for 24 h. The dead rats in each group were recorded (% dead), and the LD_50_ value was calculated according to the following equation Kärber [52].

**LD_50_ = LD_100_ − ∑ (a × b)/*n***

where LD_100_ = the lethal dose triggering 100% mortality; a = dose difference: the discrepancy between two successive doses of the administered substance; b = mean mortality: the average number of dead rats in two consecutive doses, and *n* = group population: the total number of rats per group.

### 4.5. Biological Effects of DBT, DBT–CSNPs, and Cisplatin on CCl_4_-Induced Liver Injuries

**Figure 7:**
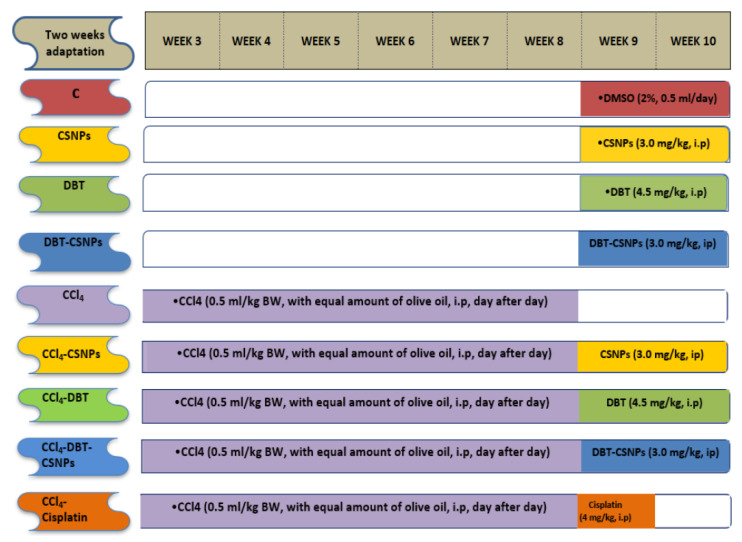
Illustration of the current experimental design.

The doses of DBT and DBT–CSNPs were chosen to be safe, with respect to their LD_50_ values where these doses approached that of cisplatin. DBT and DBT–CSNPs and CSNPs were dissolved in 2% DMSO [53,54]. Seventy-two Sprague–Dawley rats were divided into nine groups (eight rats/group) as illustrated in (Figure 7): Control group (C): rats were treated with 0.5 mL DMSO (2%) for 14 days (at 9th and 10th weeks), DBT group: animals were treated (ip) with DBT (4.5 mg/kg BW/day for two weeks (at 9th and 10th weeks), CSNPs group: rats were treated (ip) with 3.0 mg CSNPs/kg BW/day for two weeks (at 9th and 10th weeks), DBT–CSNP group: rats were treated (ip) with DBT–CSNPs (3.0 mg/kg BW/day for two weeks (at 9th and 10th weeks), CCl_4_ group: rats were injected (ip) with 0.5 mL of 99.9% CCl_4_/kg BW, with equal amounts of olive oil, day after day for six weeks [14]. CCl_4_-CSNPs, CCl_4_-DBT, CCl_4_-DBT–CSNPs groups: rats were injected with CCl_4_ for six weeks and were then treated with the same doses and periods of CSNPs, DBT, and DBT–CSNPs. CCl_4_- cisplatin: rats were injected with CCl_4_ for six weeks, and then they were treated with 4 mg of cisplatin/kg BW/day, ip, for five consecutive days [55]. At the end of the experimental period, all animals were fasted overnight, anesthetized with carbon dioxide, and then sacrificed. Blood was collected from the caudal vena cava and kept for 15 min at room temperature, after which the blood was centrifuged at 3000 rpm for 10 min, and the serum was kept at −20 °C until use. The livers were extracted directly where small portions were taken and stored in 10% formalin for the histopathological screening. The remaining livers were washed with cold saline solution (0.9% NaCl), divided into two parts, and stored at −80 °C. One of these parts was homogenized using a glass–Teflon Homogenizer in nine volumes of 0.1 M sodium phosphate buffer (pH 7.4) containing 0.9% NaCl, and the homogenate was centrifuged at 4000 rpm at 4 °C for 15 min. The supernatant was stored at −80 °C until used for evaluation of the markers of OS (MDA, GSH levels, and the activities of GPx, SOD, GST, and GR). The other part was used for the determination of the expression levels of caspase-8, Bcl-2, Bax, and DNAF.

### 4.6. Effect of the Studied Compounds on OS Markers

The level of MDA (as oxidant) and the antioxidants {GSH level and the activities of GPx (EC 1.11.1.19), GR (EC 1.8.1.7), GST (EC 2.5.1.18), and SOD (EC 1.15.1.1)} were determined according to the methods of Ohkawa, Ohishi [56] Ellman [57], Rotruck, Pope [58], Bergmeyer, Bergmeyer [59], Habig, Pabst [60], and Marklund and Marklund [61].

### 4.7. Effect of the Studied Compounds on Apoptotic Markers

#### 4.7.1. Determination of Gene Expressions of Bcl-2, Bax, and Caspase-8

Total RNA was extracted from the liver tissues using an RNA isolation kit (Thermo Scientific, Fermentas, #K0731, Waltham, MA, USA). Total RNA was measured by a NanoDrop 2000 spectrophotometer (Thermo Scientific, Waltham, MA, USA). A reverse transcription kit (Thermo Scientific, Fermentas, #EP0451) was used to produce complementary DNA (cDNA). Then, the cDNA was magnified using 2X Maxima SYBR Green/ROX qPCR Master Mix (Thermo Scientific, USA, # K0221). Table 3 shows the primers used for Bax, Bcl-2, caspase-8, and β-actin. The RT-PCR cycle parameters were 10 min at 95 °C accompanied by 40 cycles, including denaturation at 95 °C for 15 s, annealing at 60 °C for 30 s, and elongation at 72 °C for 30 s, with final elongation at 72 °C for 5 min. A StepOnePlus™ Real-Time PCR System (Applied Biosystems, Life technology (Carlsbad, CA, USA) was used for qRT-PCR. To prove the purity of the PCR products after each reaction, the division curve program was used. The critical threshold (CT) values of the target gene were normalized with quantities (CT) of a housekeeping gene (β-actin) using the 2^−∆∆Ct^ style to estimate the fold change in the target gene.

#### 4.7.2. Determination of DNAF

DNAF was determined using the agarose gel electrophoresis technique [51]. Liver tissues were homogenized in five volumes of 50 mM Tris-HCl buffer containing 50 mM EDTA and 20% sucrose, pH 7.6. The genomic DNA was separated using a DNA purification kit (G-spin^TM^ Total, Cat.No.17045, Korea). Then, 15 µg of DNA/lane was loaded and separated by electrophoresis for 2 h on 1% agarose gel containing ethidium bromide (10 µg/mL). Finally, DNA bands were visualized using trans-illumination with ultraviolet light (Consort, Turnhout, Belgium).

### 4.8. Effect of the Studied Compounds on Liver and Kidney Functions and the Lipid Profile

Liver biomarkers involving serum AST (EC 2.6.1.1), ALT (EC 2.6.1.2), and ALP (EC 3.1.3.1) activities, STP and albumin, and LTP were assayed using commercial kits (Biosystem, Barcelona, Spain) [62,63,64]. The lipid profile (serum TG, cholesterol, HDL, and LDL levels) [65,66] and kidney markers (urea and creatinine levels) were determined using kits (Biosystem, Barcelona, Spain) [67,68].

### 4.9. Liver Histopathological Analysis

Liver histopathological examinations of the various studied groups were analyzed by fixing, processing, and embedding the liver tissues in paraffin wax. Sections of 5 µm in thickness were broken and stained using hematoxylin and eosin (H & E) for examination by a light microscope [69].

### 4.10. Cell Cycle Analysis

In the different cell cycle phases (G0/G1, S and G2/M), the distribution of HepG2 cells was estimated [70]. Briefly, HepG2 cells were divided into two parts: the first part was untreated, and the second part was treated with 40 μg/mL of DBT and DBT–CSNPs separately for 24 h. All cells were then harvested and transferred to ice-cold ethanol (70%) for 12 h at 4 °C and centrifuged for 5 min at 1000 rpm. Pellets were placed in propidium iodide (0.05 mg/ mL) and RNase (100 U/mL) in phosphate buffer, pH 7.4, incubated for 30 min at 37 °C, and DNA was determined by a flow cytometer (Attune^®^ acoustic focusing flow cytometer, Thermo Scientific, Waltham, MA, USA). Then, cell cycle data were analyzed using Cellquest Software.

### 4.11. Statistical Analysis

Data were expressed as the means ± SD (standard deviation). The comparisons between individuals were obtained using Duncan’s multiple range test (DMRT) via one-way analysis of variance (ANOVA) by using SPSS, 18.0 Software, 2011 (SPSS Inc., Chicago, IL, USA). The values were estimated to be statistically significant when *p* < 0.05.

## 5. Conclusions

The LD_50_ values of DBT and DBT–CSNPs are 1350 and 1800 mg/kg, respectively. DBT and DBT–CSNPs have a therapeutic effect against CCl_4_-induced liver injuries via the reduction of apoptosis. DBT-CSNPs have a greater effect than DBT, and both compounds have a greater effect than cisplatin. Additionally, DBT and DBT–CSNPs have antineoplastic activities against the HepG2 cell line. Low doses of DBT and DBT–CSNPs for 14 days have no adverse effect in healthy rats. Therefore, further research must be done on DBT–CSNPs and DBT to identify their clinical implementations.

## Figures and Tables

**Table 1:** Karber’s method for the determination of the LD_50_ of DBT and DBT–CSNPs in rats.

Groups	Dose (mg/kg)	Dose Difference (a)	No. of Rats	LD_50_ of DBT			LD_50_ of DBT–CSNPs		
				No. of Dead	Mean Mortality (b)	Product (a × b)	No. of Dead	Mean Mortality (b)	Product (a × b)
**1**	**200**	-	4	0	-	-	0	-	-
**2**	400	200	4	1	0.5	100	0	-	-
**3**	800	400	4	1	1	400	1	0.5	200
**4**	1200	400	4	2	1.5	600	1	1	400
**5**	2000	800	4	3	2.5	2000	2	1.5	1200
**6**	3000	1000	4	4	3.5	3500	4	3	3000
**Sum of product**						6600			4800
**LD_50_**				LD_50_ = 3000 ‒ (6600/4) = 1350 mg/kg			LD_50_ = 3000 − (4800/4) = 1800 mg/kg		

**Table 2:** Effect of different compounds on the liver, kidney functions, and lipid profile.

Parameters	C	CSNPs	DBT	DBT–CSNPs	CCl_4_	CCl_4_-CSNPs	CCl_4_-DBT	CCl_4_-DBT–CSNPs	CCl_4_-Cisplatin
**ALT (U/L)**	117.72 ± 2.54	118.3 ± 2.56	120.58 ± 3.74	122.5 ± 2.81	265.52 ± 4.33 *	258.31 ± 3.89 *^α^	188.33 ± 2.74 *^#α^	157.92 ± 6.09 *^#^	215.8 ± 6.8 *^#α^
**AST (U/L)**	146.19 ± 3.86	148.3 ± 3.69	152.78 ± 5.4	150.7 ± 4.7	302.1 ± 4.27 *	293.61 ± 3.89 *^α^	224.1 ± 3.25 *^#α^	187.49 ± 4.14 *^#^	259.58 ± 6.07 *^#α^
**ALP (U/L)**	252.85 ± 2.08	254.25 ± 2.09	257.8 ± 4.1	255.85± 4.29	456.31 ± 5.58 *	446.49 ± 5.41 *^α^	352.16 ± 4.05 *^#α^	314.9 ± 3.44 *^#^	375.2 ± 5.72 *^#α^
**TP (g/dl)**	5.89 ± 0.042	5.87 ± 0.037	5.80 ± 0.035	5.84 ± 0.037	4.99 ± 0.033 *	5.06 ± 0.039 *^α^	5.57 ± 0.045 *^#^	5.67 ± 0.035 *^#^	5.08 ± 0.095 *^α^
**Liver TP (mg/g tissue)**	143.1 ± 7.38	143.6 ± 3.92	139.8 ± 4.34	141.2 ± 3.77	91.03 ± 5.88 *	87.21 ± 5.22 *^α^	105.2 ± 7.15 *^#^	121.1 ± 5.23 *^#^	83.5 ± 4.75 ^*α^
**Albumin (g/dL)**	4.99 ± 0.33	4.91 ± 0.28	4.88 ± 0.063	4.84 ± 0.057	3.48 ± 0.15 *	3.57 ± 0.14 *^α^	3.91 ± 0.025 *^#^	3.77 ± 0.057 *^#^	3.35 ± 0.054 *^α^
**Cholesterol (mg/dL)**	88.75 ± 2.39	90.3± 2.31	91.17± 3.98 *	93.08 ± 2.16	165.44 ± 2.32 *	160.7 ± 4.21 *^α^	131.11 ± 2.61 *^#^	128.94 ± 2.57 *^#^	153.78 ± 2.39 *^#α^
**TG (mg/dL)**	87.49 ± 1.58	89.2 ± 1.62	90.08 ± 2.16	92.07 ± 2.8	148.03 ± 2.27 *	145.9 ± 3.26 *^α^	128.75 ± 1.67 *^#α^	123.84 ± 2.78 *^#^	133.3 ± 1.9 *^#α^
**LDL–cholesterol** **(mg/dL)**	55.61 ± 1.5	56.69 ± 1.54	61.19 ± 1.96	58.69 ± 1.56	93.1± 1.87 *	90.2 ± 2.83 *^α^	78.28 ± 2.2 *^#α^	70.79 ± 1.45 *^#^	86.59 ± 2.01 *^#α^
**HDL–cholesterol (mg/dL)**	47.61 ± 1.45	45.2 ± 1.42	44.37 ± 1.51	42.88 ± 1.86	27.95 ± 2.09 *	28.9 ± 1.93 *^α^	40.22 ± 1.17 *^#^	41.42 ± 0.87 *^#^	33.74 ± 1.74 *^#α^
**Urea (mg/dL)**	43.79 ± 2.16	45.2 ± 2.19	46.74 ± 2.35 *	45.07 ± 3.96	73.21 ± 2.98 *	71.6 ± 1.09 *^α^	65.76 ± 3.19 *^#α^	58.74 ± 2.35 *^#^	69.74 ± 2.26 *^α^
**Creatinine (mg/dL)**	0.95 ± 0.02	0.97 ± 0.13	0.99 ± 0.108	1.03 ± 0.04	1.5 ± 0.068 *	1.42 ± 0.071 *^α^	1.38 ± 0.049 *^#α^	1.21 ± 0.027 *^#^	1.44 ± 0.04 *^α^

Values represent the mean ± SD of eight rats. One-way ANOVA followed by Tukey’s test was used (* *p* < 0.05 versus saline control group, # *p* < 0.05 versus CCl_4_ group & α *p* < 0.05 versus DBT–CSNP-treated group).

**Table 3:** Primers used in the synthesis of cDNA.

Gene	Forward Primer (5′ ------ 3′)	Reverse Primer (5′ ------ 3′)
***Bcl-2***	ATCGCTCTGTGGATGACTGAGTAC	AGAGACAGCCAGGAGAAATCAAAC
***Bax***	ACACCTGAGCTGACCTTG	AGCCCATGATGGTTCTGATC
***Caspase-8***	CTGGGAAGGATCGACGATTA	CATGTCCTGCATTTTGATGG
***β-actin***	AAGTCCCTCACCCTCCCAAAAG	AAGCAATGCTGTCACCTTCCC

## Data Availability

All data generated or analyzed during this study are included in this published article.

## Abbreviations

Apaf-1	apoptosis protease activating factor-1
CCl_4_	carbon tetrachloride
^•^CCl_3_	trichloromethyl radical
CCl_3_OO^•^	trichloromethylperoxy radicals
CS	chitosan
CSNPs	chitosan nanoparticles
DBT	titanium (IV)-thiophenolate complex
DBT–CSNPS	titanium (IV)–dithiophenolate -Chitosan nanocomposite
DMSO	Dimethyl sulfoxide
DTP	dithiophenolato ligand
GPx	total glutathione peroxidase
GR	glutathione reductase
GSH	reduced glutathione
GST	glutathione-S-transferase
GSSG	oxidized glutathione
HepG2	human liver cancer
MDA	malondialdehyde
RNS	reactive nitrogen species
ROS	reactive oxygen speci
SOD	superoxide dismutase
THLE2	normal liver cell
